# Structural and molecular biology of hepatitis E virus

**DOI:** 10.1016/j.csbj.2021.03.038

**Published:** 2021-04-07

**Authors:** Bo Wang, Xiang-Jin Meng

**Affiliations:** Center for Emerging, Zoonotic and Arthropod-borne Pathogens, Department of Biomedical Sciences and Pathobiology, Virginia-Maryland College of Veterinary Medicine, Virginia Polytechnic Institute and State University, Blacksburg, VA, USA

**Keywords:** Hepatitis E Virus (HEV), Genetic Diversity, Virion structure, Genomic organization, Proteins and functions, Life cycle of HEV

## Abstract

Hepatitis E virus (HEV) is one of the most common causes of acute viral hepatitis, mainly transmitted by fecal-oral route but has also been linked to fulminant hepatic failure, chronic hepatitis, and extrahepatic neurological and renal diseases. HEV is an emerging zoonotic pathogen with a broad host range, and strains of HEV from numerous animal species are known to cross species barriers and infect humans. HEV is a single-stranded, positive-sense RNA virus in the family *Hepeviridae*. The genome typically contains three open reading frames (ORFs): ORF1 encodes a nonstructural polyprotein for virus replication and transcription, ORF2 encodes the capsid protein that elicits neutralizing antibodies, and ORF3, which partially overlaps ORF2, encodes a multifunctional protein involved in virion morphogenesis and pathogenesis. HEV virions are non-enveloped spherical particles in feces but exist as quasi-enveloped particles in circulating blood. Two types of HEV virus-like particles (VLPs), small *T* = 1 (270 Å) and native virion-sized *T* = 3 (320–340 Å) have been reported. There exist two distinct forms of capsid protein, the secreted form (ORF2^S^) inhibits antibody neutralization, whereas the capsid-associated form (ORF2^C^) self-assembles to VLPs. Four *cis*-reactive elements (CREs) containing stem-loops from secondary RNA structures have been identified in the non-coding regions and are critical for virus replication. This mini-review discusses the current knowledge and gaps regarding the structural and molecular biology of HEV with emphasis on the virion structure, genomic organization, secondary RNA structures, viral proteins and their functions, and life cycle of HEV.

## 1 Introduction

Hepatitis E was first recognized in the 1980 s as a ‘non-A, non-B hepatitis’ associated with waterborne outbreaks in India. The causative agent, hepatitis E virus (HEV), was identified using immune electron microscopy, and the viral genomic sequence was determined in 1990 [1]. HEV is one of the most common causes of acute viral hepatitis worldwide. Globally, there are an estimated 20 million HEV infections, leading to 3.3 million symptomatic cases of hepatitis E and approximately 44,000 hepatitis E-associated deaths in 2015 [2], [3]. In developing countries with poor sanitation conditions, HEV transmits primarily to humans via the fecal-oral route through drinking feces-contaminated water. In industrialized nations, sporadic and cluster cases of hepatitis E are reported due to ingestion of raw or undercooked animal meat products [4]. Although acute hepatitis E rarely progresses to acute liver failure or chronicity, HEV infections in pregnant women have a high incidence of developing fulminant hepatic failure with a case-fatality rate of up to 30% [5]. Furthermore, chronic hepatitis E has become a significant clinical problem since the majority of HEV infections in immunosuppressed individuals can progress into chronicity which requires antiviral treatment, and otherwise chronic infection leads to cirrhosis that needs transplantation [6]. Ribavirin is a reasonably effective antiviral for patients chronically infected with HEV, with sustained virological response (SVR) of approximately 85%; however, a minority of patients fail to achieve SVR, possibly because of viral mutants [7]. In addition to acute and chronic hepatitis, HEV infection is also associated with a wide range of extrahepatic manifestations such as neurological and renal injuries [8]. Notwithstanding the above, an HEV-specific antiviral is still lacking, and a vaccine against HEV is available only in China [9].

Since the initial discovery of zoonotic HEV in 1997 from domestic pigs in the United States [10], HEV is now recognized as an important emerging zoonotic pathogen with a large number of animal reservoirs, including swine, deer, rabbit, camel, and rat. Also, increasingly diverse strains of HEV have been identified in numerous animal species, although their host range and pathogenicity are mostly unknown [11].

Tremendous progress has been made in the biological and structural characterization of HEV. The determination of the high-resolution three-dimensional structure of HEV virus-like particle (VLP) and capsid protein helps understand HEV morphogenesis and pathogenesis [12]; the development of various reverse genetic systems for HEV allows to delineate the structural and functional relationship of HEV genes [13]; the establishment of more efficient cell culture systems and relevant animal models for HEV provided tools to understand the molecular mechanisms of HEV life cycle and virus-host interactions [2]. In this mini review, we highlight the recent advances that unveil the structural and molecular biology of HEV.

## 2 Virus taxonomy and genetic diversity

**Fig. 1:**
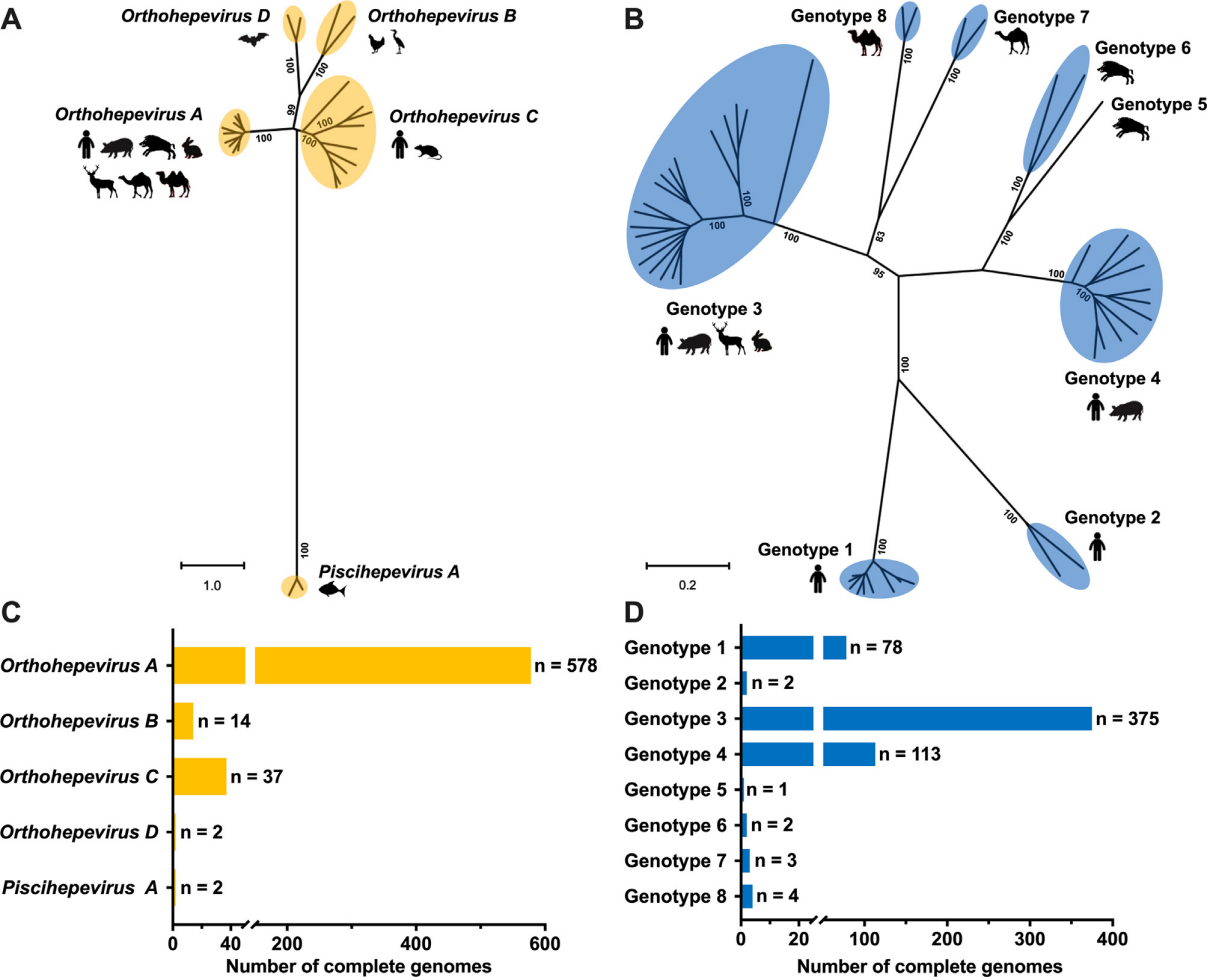
The taxonomy and genetic diversity of HEV. (A) A maximum-likelihood tree based on the complete genomes of representative members of the family *Hepeviridae*. HEV classification of five species within the two genera is shown according to the International Committee on Taxonomy of Viruses (ICTV) consensus proposal. The major host tropism of each virus species is indicated by animal icons. GenBank accession numbers of representative viral strains used: species *Orthohepevirus A* (M73218, KX578717, AB301710, FJ906895, AB197673, AB573435, AB602441, KJ496143, and KX387865); species *Orthohepevirus B* (AY535004 and KX589065); species *Orthohepevirus C* (GU345042, JN998606, KY432899, KY432901, KY432902, MG020022, MG020024, MG021328, and MK192405); species *Orthohepevirus D* (JQ001749 and KX513953); species *Piscihepevirus A* (MN995808 and HQ731075). Complete genomes are aligned using the MAFFT algorithm in Geneious Prime software version 2021.0.3. Evolutionary analyses are conducted in Molecular Evolutionary Genetics Analysis Software X (MEGA X) version 10.1.7 with 1,000 bootstrap reiterations. General Time Reversible (GTR) + Gamma Distributed (G) nucleotide substitution model with the lowest Bayesian Information Criterion (BIC) score was selected based on Find Best-Fit Substitution Model (ML) in MEGA X. Bootstrap values (>80%) are indicated at specific nodes. Bars indicate the number of nucleotide substitutions per site. (B) A maximum-likelihood tree based on the complete genomes of representative members of the species *Orthohepevirus A*. The eight different genotypes are shown according to the ICTV consensus proposal. The major host tropism of each genotype is indicated by animal icons. GTR + G + Invariable Sites (I) nucleotide substitution model with the lowest BIC score was selected. GenBank accession numbers of representative viral strains used are: Genotype 1 (FJ457024, MH918640, M73218, L08816, X98292, AY230202, AY204877, JF443721, LC225387); Genotype 2 (KX578717 and MH809516); Genotype 3 (AB290313, KP294371, LC260517, MF959764, MF959765, MK390971, AF082843, AP003430, FJ705359, AB248521, AB369687, AF455784, JQ013794, FJ998008, AY115488, AB369689, JQ953664, KU513561 and, FJ906895); Genotype 4 (AB369688, MK410048, AB197673, DQ279091, AB074915, AJ272108, AY723745, AB220974, AB108537, GU119961, and AB369690); Genotype 5 (AB573435); Genotype 6 (AB856243 and AB602441); Genotype 7 (KJ496144 and KJ496143); Genotype 8 (MH410174 and KX387865). (C) Complete genomes of five species within the two genera in the family *Hepeviridae*. Numbers of genomic sequences of each species are indicated. (D) Complete genomes of eight different genotypes within the species *Orthohepevirus A*. Numbers of genomic sequences of each genotype are indicated. Complete genomes analyzed in this study are acquired in GenBank (retried as of January 2021).

According to the 10th International Committee on the Taxonomy of Viruses (ICTV) Report, HEV is classified in the family *Hepeviridae*, which contains two distinct genera: *Orthohepevirus* and *Piscihepevirus*. The former genus contains four species (*Orthohepevirus A* to *D*), whereas the latter contains a single species (*Piscihepevirus A*) [14] (Fig. 1A). The species *Orthohepevirus A* is divided into at least eight distinct genotypes: genotypes 1 and 2 infect only humans and cause large waterborne outbreaks in endemic regions of South and Southeast Asia, Africa, and Mexico; genotypes 3 and 4 infect a wide range of mammals, including humans, swine, deer, and rabbits, and cause sporadic cases of hepatitis E in comparably developed countries of Europe and East Asia; genotypes 5 and 6 were identified from wild boars in Japan; genotype 7 from dromedaries in Middle East countries; genotype 8 from Bactrian camels in China (Fig. 1B). *Orthohepevirus B* and *D species* include viruses from birds and bats, respectively, and *Orthohepevirus C* species from rats, shrews, ferrets, minks, and wild rodents [15]. Although genotypes 3 and 4 HEVs from swine are the main sources of zoonotic infection in humans, animal strains of genotypes 5, 7, and 8 HEVs from species *Orthohepevirus A* and rat HEVs from species *Orthohepevirus C* are also known to have zoonotic potential [11], [16]. Additional strains of distantly-related HEVs remain unclassified due to the lack of complete genomes or ambiguous phylogenetic position [14]. With the ever-expanding host range and identification of genetically divergent HEV strains, the taxonomy of the family *Hepeviridae* will continue to evolve.

The genome of HEV shows striking diversity and sequence variation. Most of the 600 full-length HEV genomes available in the GenBank database belong to species *Orthohepevirus A* (Fig. 1C). Based on the host species, geographical origin, phylogenetic relationship, and clinical outcome, the species *Orthohepevirus A* consists of at least eight genotypes and 36 subtypes so far. According to the ICTV, a subtype assignment requires at least three complete viral genomes that are phylogenetically distinct from previous strains and epidemiologically unrelated. Therefore, multiple divergent HEV strains are still unassigned due to the fewer than three complete genome sequences [17]. To perform enhanced molecular typing and epidemiological investigations specifically to HEV, an HEV website (HEVnet) was established in 2017 (https://www.rivm.nl/mpf/typingtool/hev/) [18]. Genotypes 3 and 4 HEVs from diverse animals exhibit remarkable genetic heterogeneity. Notably, zoonotic genotypes 3 and 4 HEVs cause chronic HEV infections in immunocompromised individuals as well as extrahepatic diseases. As of January 2021, nearly 400 genotype 3 HEV genomic sequences have been identified (Fig. 1D). In addition to the unique transmission pattern and clinical course of different genotypes, the relationship between HEV genetic variability and liver disease status or resistance to antivirals has been investigated. Several mutations in the viral polymerase of genotype 3 HEV are reportedly associated with ribavirin treatment failure in organ transplant recipients. For example, the Y1320H and G1634R mutations enhanced viral fitness, and the K1383N mutation suppressed viral replication but increased ribavirin susceptibility [19], [20], [21].

## 3 Virion structure

**Fig. 2:**
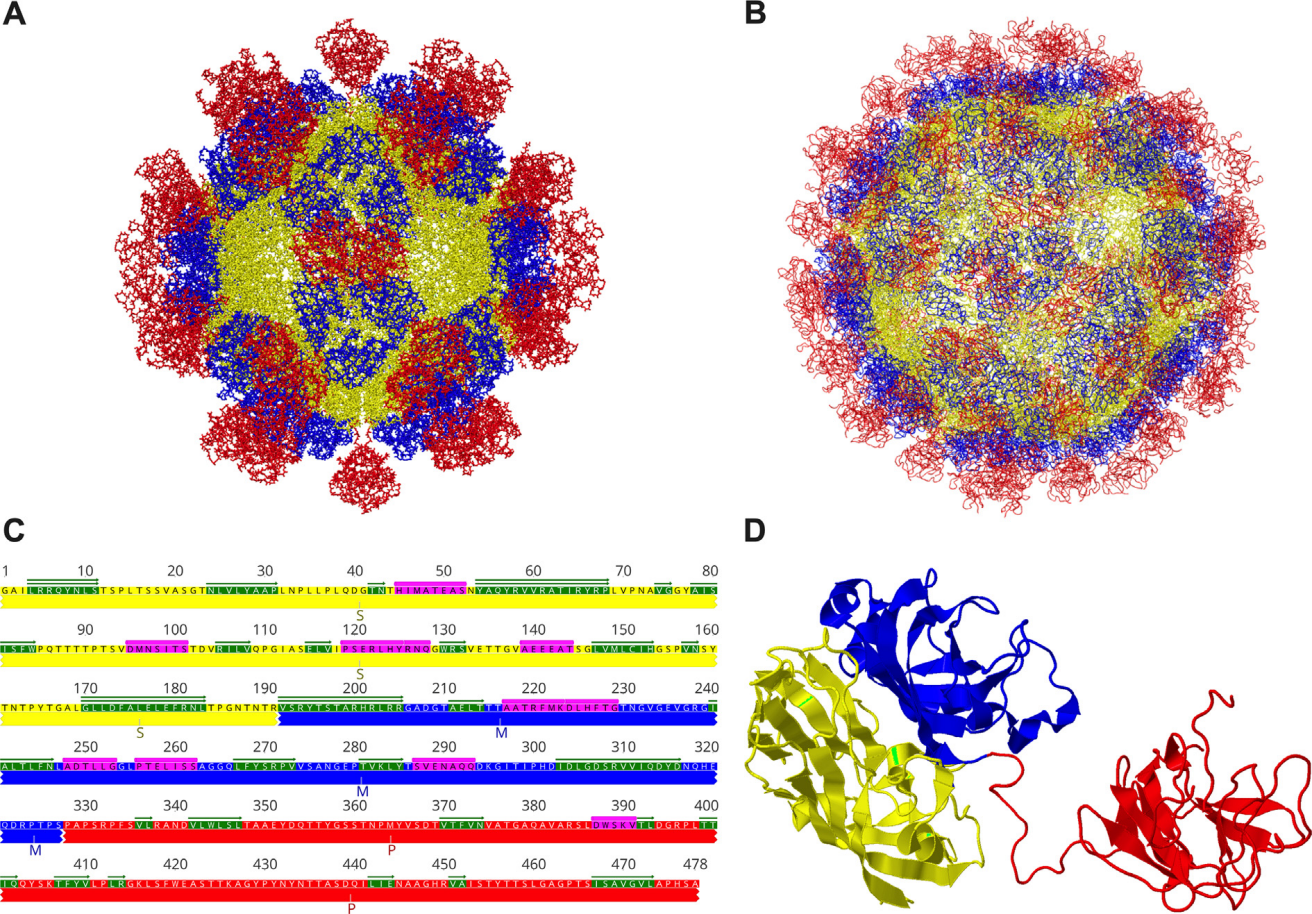
Structure interpretation of hepatitis E virus. (A) *T* = 1 HEV virus-like particle (VLP) (PDB accession no. 2ZTN) comprises 60 capsid subunits. (B) *T* = 3 HEV VLP (PDB accession no. 3IYO) is composed of 180 capsid subunits. HEV VLP structures are generated in 3D viewer software Cn3D version 4.3.1. (C) Representation of secondary structure assignment of HEV truncated capsid protein. The ORF2 sequence corresponds to amino acid residues 119 to 606 of the HEV prototype Burma strain (GenBank accession no. M73218). The S (shell), M (middle), and P (protruding) domains are shown in yellow, blue, and red, respectively. α-helices and β-sheets are indicated in pink and green, respectively. (D) Secondary structure of HEV truncated capsid protein shows S, M, and P domains at the left, middle, and right, respectively. Modified from various studies [28], [29]. (For interpretation of the references to colour in this figure legend, the reader is referred to the web version of this article.)

HEV virions exist in two forms in the infected host, non-enveloped (neHEV) and quasi-enveloped (eHEV) particles [22]. Virions secreted in feces are non-enveloped, spherical particles of approximately 27–34 nm in diameter. However, virions secreted in circulating blood and supernatant of infected cell cultures are quasi-enveloped as they are covered with a lipid envelope [23]. Although neHEV particles are more infectious, eHEV particles are resistant to antibody neutralization against the viral capsid protein [24]. The HEV capsid proteins assemble into virion particles, binds host cells, and elicit neutralizing antibodies. Expression of a truncated capsid protein in insect cells by baculovirus expression system resulted in the self-assembly of capsid protein and production of two types of virus-like particles (VLPs): the small *T* = 1 (270 Å in diameter) (Fig. 2**A**) and native virion-sized *T* = 3 VLPs (320–340 Å) (Fig. 2B). It has been demonstrated that amino acid residues 126 to 601 are the essential elements required for the *T* = 1 VLPs assembly. In contrast, amino acids 14–608 including the signal sequence and N-terminal arginine-rich region are necessary for the *T* = 3 VLPs formation. Both *T* = 1 and *T* = 3 VLPs are icosahedral but consist of different copies of the truncated capsid protein: 60 subunits for *T* = 1 and 180 subunits for *T* = 3 [12], [25], [26]. The production of the *T* = 1 VLPs is caused by deleting the N-terminal basic domain of the capsid protein and forms empty particles with no viral RNA inside [27]. Despite variations in some amino acid residues, the crystal structure of *T* = 1 VLPs of genotypes 1, 3, and 4 HEVs are almost identical [25], [28], [29]. The cryoelectronic microscope structure of *T* = 3 VLPs has also been resolved [25].

Interactions of dimeric, trimeric, and pentameric capsid subunits around respective two-, three-, five-fold icosahedral axes have been demonstrated for *T* = 1 VLP packaging. The crystal structure of *T* = 1 VLP resolved at the resolution of amino acid level reveals that each capsid monomer contains three distinct domains, S (shell), M (middle), and P (protruding) (Fig. 2C). The S domain is composed of jelly roll-like β-sheets; the M domain is tightly linked to the S domain and locates at the surface around the three-fold axis of the particle; the P domain dimerizes forming protruding spikes around the two-fold axis of *T* = 1 VLP and interacts with host cells [25], [29], [30] (Fig. 2D). A flexible proline-rich hinge region linked between M and P domains contributes to the topological changes of both *T* = 1 and *T* = 3 VLP [12], [25]. An *in vitro* assembly assay indicated that the pentamer formation is preferable, and mutagenesis analyses showed that the Tyr288 residue in the center of the pentamer was highly conserved and crucial for particle formation [29]. Substitutions of amino acid residues from 489 to 586, located at the exposed loops of the apical center region of the protruding spike, significantly reduced HEV attachment activity to its susceptible cells, indicating that this particular region is involved in receptor binding and neutralization antibody recognition [29]. It has been shown that the immunogen p239 (residues 368–606) in the Hecolin vaccine covering the partial M domain and the whole P domain forms a shrunken version of the *T* = 1 VLPs and contains functional HEV immune epitopes [31]. Although the capsid protein contains three potential N-glycosylation sites (Asn137, Asn310, and Asn562), there is a lack of a signal peptide-like sequence in the VLPs, thus the glycosylation process is likely not required for assembling infectious particles [32]. Notably, it has been recently validated that the capsid protein for virion packaging has no glycosylation, but the secreted ORF2 product is glycosylated [33], [34]. The available structural information about the HEV capsid protein provided important molecular insights into viral assembly and entry, and will aid in future vaccine design and antiviral development.

## 4 Genomic organization and secondary RNA structures

**Fig. 3:**
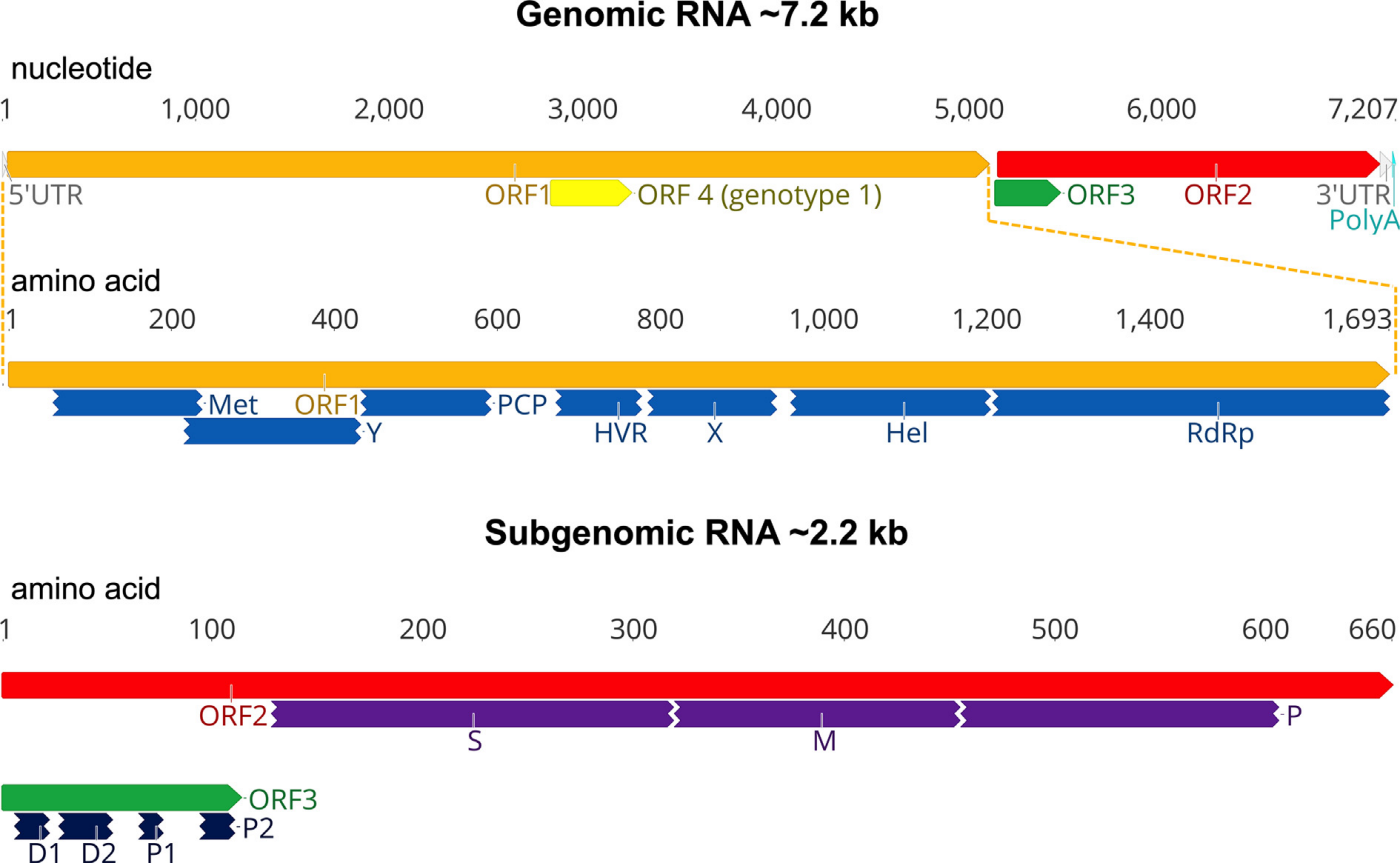
A schematic diagram of the genomic organization of hepatitis E virus (HEV). The HEV genomic RNA is approximately 7.2 kb in length, comprising a short 5′ untranslated region (UTR), three partially overlapping open reading frames (ORFs), and a 3′ UTR. The 5′ UTR contains a 7-methylguanosine cap (7mG), and the 3′ UTR is polyadenylated (polyA). ORF1 encodes the non-structural polyprotein, including multiple functional domains: methyltransferase (Met), Y domain, papain-like cysteine protease (PCP), hypervariable region (HVR), X domain, helicase (Hel), and RNA-dependent RNA polymerase (RdRp). ORF2 encodes the structural capsid protein, containing S (shell), M (middle), and P (protruding) domains. ORF3 overlaps partially with ORF2 and encodes a multifunctional protein harboring two hydrophobic domains (D1 and D2) and two proline-rich domains (P1 and P2). An additional novel ORF4 has been identified solely in genotype 1 HEV. ORF2 and ORF3 are translated from a bicistronic 2.2 kb subgenomic mRNA (sgRNA) generated during viral replication. The nucleotide positions are according to the HEV prototype Burma strain (GenBank accession no. M73218). The genomic RNA in nucleotide bases and ORFs in amino acids are shown on the top.

Apart from the *Orthohepevirus A* species, the genomic features of other HEV have not been well characterized. HEV genomes within *Orthohepevirus A* are single-stranded, positive-sense RNA of ~ 7.2 kb in length, comprising a short 5′ untranslated region (UTR), three partially overlapping open reading frames (ORFs), and a 3′ UTR [14] (Fig. 3). For HEV coding regions, the ORF1 encodes a non-structural polyprotein with multiple potentially functional domains: methyltransferase (Met), Y domain, papain-like cysteine protease (PCP), hypervariable region (HVR), X domain, helicase (Hel), and RNA-dependent RNA polymerase (RdRp). It is still debatable whether the ORF1 polyprotein undergoes processing into individual functional protein. The ORF2 encodes the structural capsid protein, which contains the S, M, and P domains. The ORF3 overlaps partially with ORF2 and encodes a multifunctional phosphoprotein harboring two hydrophobic domains (D1 and D2) and two proline-rich domains (P1 and P2). Recently, a novel ORF4 has been identified in genotype 1 HEV, but not in other HEV genotypes, and endoplasmic reticulum (ER) stress promotes viral replication by inducing translation of the novel ORF4 in genotype 1 HEV [35].

**Fig. 4:**
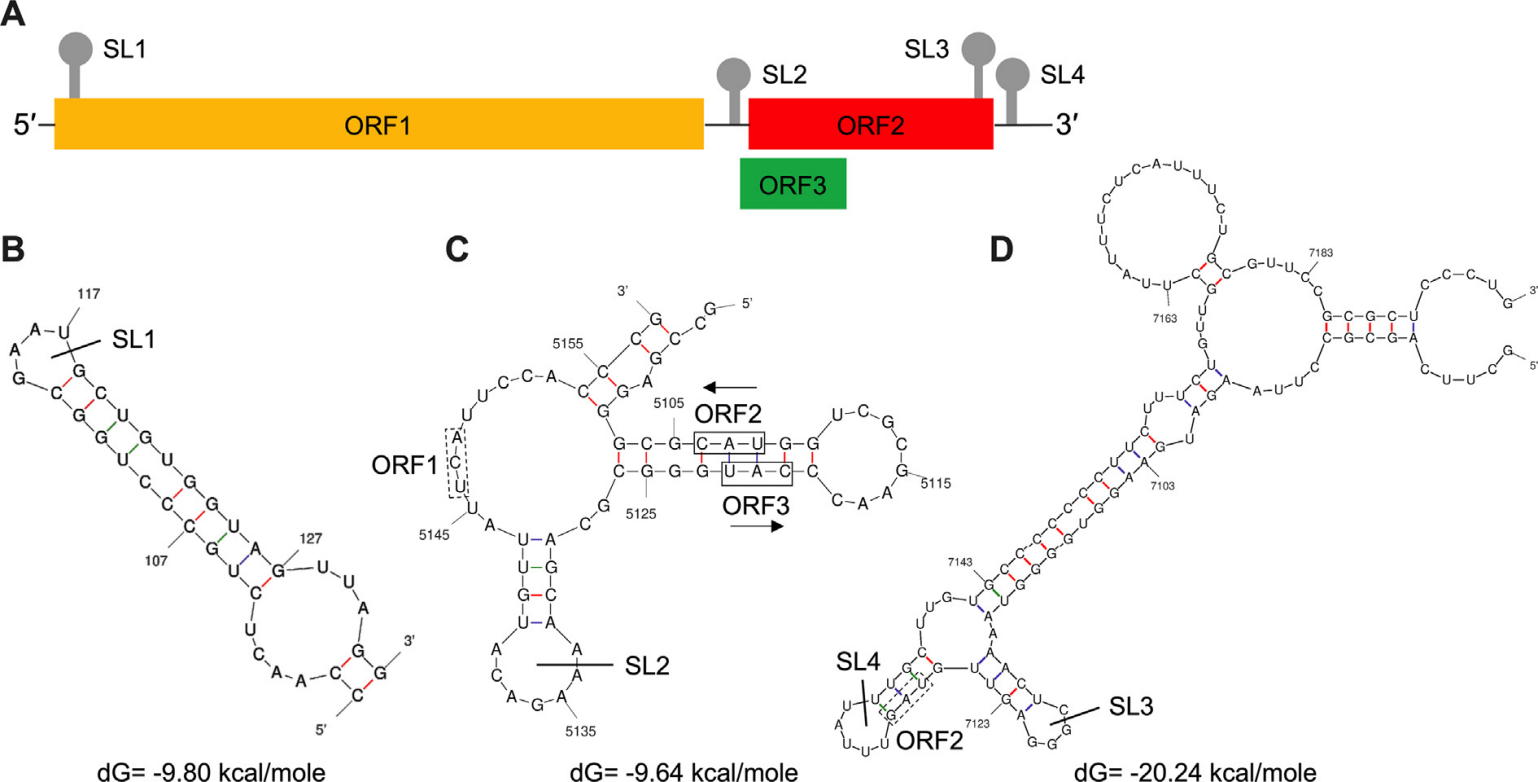
RNA stem-loop (SL) structures of *cis*-reactive elements (CREs) in hepatitis E virus (HEV) genome. The RNA secondary structures are predicted using the Unified Nucleic Acid Folding and hybridization package (UNAFold). The nucleotide positions are according to the HEV prototype Burma strain (GenBank accession no. M73218) (A) Organization of the HEV genome. The locations of the four predicted SL secondary structure are depicted. (B) Predicted secondary structure at the N-terminus of ORF1 with the SL1 indicated here. The sequence corresponds to nucleotide positions 98 to 132. (C) Predicted SL and secondary structure at the junction region (JR) of the negative-polarity complementary sequence with SL2 indicated here. The sequence corresponds to nucleotide positions 5098 to 5159. The stop codon of ORF1 and start codons of ORF2 and ORF3 are labeled with boxes of solid and dot lines, respectively. (D) Predicted secondary structure at the C-terminus of ORF2 indicated here with SL3, and the 3′ UTR indicated here with SL4. The sequence corresponds to nucleotide positions 7084 to 7194. The stop codon of ORF2 is labeled with a box of dot line.

For HEV non-coding regions, the 5′ UTR contains a 7-methylguanosine cap structure (7mG), which is essential for the initiation of HEV replication and infectivity [36], [37]. The 3′ UTR is polyadenylated (polyA), and the U-rich region in the 3′ UTR poly-A tail acts as a potent pathogen-associated motif pattern (PAMP) for retinoic acid-inducible gene I (RIG-I) [38]. The ORF2 protein and ORF3 protein are expressed from a ~ 2.2 kb bicistronic subgenomic mRNA (sgRNA) [39] (Fig. 3). A stem-loop structure has been identified in the junction region (JR) between the end of ORF1 and the start of ORF3, which is crucial for sgRNA transcription and synthesis [40], [41]. At least four *cis*-reactive elements (CREs) containing stem-loops from secondary RNA structures have been identified in the HEV genome (Fig. 4): one locates between the end of ORF2 and 3′ UTR [36], another between the end of JR and the beginning of ORF3 [42], the remaining two locate at the start of ORF1 and at the end of ORF2 coding region [43]. These CREs are highly conserved across different HEV genotypes and critical for HEV replication. Future studies are warranted to define the underlying mechanism of these non-coding regions, especially the stem-loop structures, in regulating HEV replication.

## 5 Viral Proteins, their primary structural features and functions

### 5.1 Nonstructural polyprotein encoded by ORF1

Putative functional domains, including Met, PCP, Hel, and RdRp, have been predicted through computer-assisted sequence comparison of HEV ORF1 polyprotein with proteins of other positive-strand RNA viruses [44]. Whether ORF1 polyprotein is processed during HEV replication remains disputable. It was reported that ORF1 polyprotein is not subjected to specific proteolytic processing, which is uncommon for animal positive-stranded RNA viruses [45]. However, cleavage of both ORF1 polyprotein and ORF2 protein by the purified PCP expressed from *E. coli* cells has also been reported [46]. Nevertheless, some of these predicted functional domains within ORF1 polyprotein have now been experimentally confirmed.

The Met domain at the 5′ end of ORF1 polyprotein possesses guanine-7-Met and guanyl transferase activities [47]. The function of the PCP domain for protease activity is still debatable, although it does share a high sequence identity to the protease of the rubella virus [44]. PCP is reportedly involved in HEV immune evasion by acting as potential interferon (IFN) antagonist and possessing deubiquitinase activity for both RIG-I and TANK-binding kinase 1 (TBK-1) [48]. A plethora of positive-stranded RNA viruses encode RNA helicase and RNA-dependent RNA polymerase (RdRp), which are essential for viral replication. Similarly, HEV Hel possesses NTPase and 5′ to 3′ RNA duplex-unwinding activates. The Hel domain is also involved in RNA 5′-triphosphatase activity that catalyzes the first step of RNA capping [49]. HEV RdRp is likely located in the ER and binds the 3′ UTR of HEV genomic RNA and synthesizes the complementary strand RNA. The RdRp domain contains a GDD motif essential for HEV replicase activity [50], [51]. Certain RdRp mutations discovered in patients chronically infected with HEV have reportedly altered viral virulence, pathogenesis, and antiviral sensitivity, as described earlier.

Three additional domains within ORF1 polyprotein have also been identified, but their functions remain mostly elusive. The Y domain maps between the Met and PCP domains. Sequence analyses suggested that the Y domain is an extension of the Met domain, and mutational analyses showed its severely impaired effect on viral replication and virion infectivity [52], [53]. The HVR has a high degree of sequence variability and a large number of proline residues, which leads to an unstable tertiary structure. This intrinsically disordered HVR may regulate viral transcription and translation by facilitating the binding of HVR to a variety of ligands, enzymes, and metal ions [54], [55]. Mutational analysis of HVR has revealed its involvement in the efficiency of viral replication [56]. Moreover, HVR participates in the HEV adaptation. A naturally-occurring genotype 3 HEV recombinant with an insertion of human ribosomal protein sequence S17 in the HVR acquired an enhanced viral fitness and an expanded host range *in vitro* [57], [58]. The function of the X domain is also unclear, but it may associate with ADP-ribose-1′′-monophosphatase catalytic activities [59]. The X domain interacts with both Met domain and ORF3 protein and forms a viral replication complex [60]. The X domain serves as another putative IFN antagonist, which inhibits phosphorylation of interferon regulatory factor (IRF-3) and blocks the interferon beta (IFN-β) production [48].

### 5.2 Structural capsid protein encoded by ORF2

The N-terminus of the capsid protein contains a signal peptide sequence, which translocates the capsid protein into the ER. The capsid protein self-assembles into VLPs when expressed in recombinant baculovirus system or *E. coli* system [26]. The C-terminus of the capsid protein is essential for encapsidation of viral genome and particle stabilization [61]. The capsid protein is abundantly located in the cytoplasm and binds explicitly to the 5′ region of HEV genome [62], [63]. Recently, HEV capsid protein has been reported to be widely distributed in subcellular organelles, including the ER, Golgi, and even nucleus [64]; however, independent confirmation is still lacking. Large amounts of capsid protein are produced during HEV infection, with the majority present in the supernatant of cells or patient sera not associated with virus particles; only a minority of the capsid protein is assembled into infectious virions. There exist two distinct forms of capsid protein translated from the ORF2: a secreted form (ORF2^S^) initiated at the originally recognized ORF2 start codon and a capsid-associated form (ORF2^C^) initiated at an internal ORF2 start codon, that is 15 amino acids downstream from the ORF2^S^ start [33], [34]. ORF2^S^ undergoes post-translational modifications and has a larger protein size of 84 kDa, compared to 72 kDa for the ORF2^C^. The ORF2^S^ is implicated in HEV immune evasion by inhibiting antibody-mediated neutralization [34] but further in-depth study is warranted to delineate the precise functions of ORF2^S^.

The capsid protein is highly immunogenic and elicits neutralizing antibodies; the amino acid residues between 578 and 607 of a genotype 1 HEV contain linear and/or conformational epitopes, important for monoclonal antibody (mAb) recognition [65]. The Arg512 residue appears to be a crucial residue for interaction with a mAb 8C11 [66]. The amino acid residues Leu377 and Leu613 of a genotype 4 HEV are critical in forming neutralizing epitope [67]. Antigenic variations have been observed using strain-specific or genotype-specific mAb’s against different HEV strains [66]. Also, cross-genotype neutralizing mAb 8G12 identified several conserved amino acid residues for both genotypes 1 and 4 HEV neutralization, hinting at the existence of pan-genotypic epitopes [68]. The potential role of capsid protein in HEV pathogenicity is unknown, but three amino acid mutations (F51L, T59A, and S390L) in the capsid protein have been shown to collectively contribute to virus attenuation [69].

The commercial Hecolin HEV vaccine, based on a recombinant *E. coli*-expressed capsid protein p239, has demonstrated its safety and efficacy in a large-scale phase III clinical trial [70] and is approved and licensed for use in China in 2012 [71]. Furthermore, the Hecolin vaccine is currently undergoing a phase IV clinical trial to assess the safety, immunogenicity, and effectiveness in pregnant women in rural areas of Bangladesh [72].

### 5.3 A multifunctional protein encoded by ORF3

The ORF3 almost entirely overlaps with ORF2 and encodes a small phosphoprotein, which is phosphorylated at the Ser71 residue by the extracellularly regulated kinase (ERK), a member of the mitogen-activated protein kinase (MAPK) family [73], [74]. It appears that ORF3 protein is not required for HEV replication in hepatoma cells, but is indispensable for HEV infection in both rhesus macaques and pigs [42], [75], [76]. Remarkably, a study has shown that phosphorylation of ORF3 protein is not necessary for viral replication or infectious virions production [42]. The phosphorylation status of ORF3 protein remains to be elucidated. The filamentous and punctate distribution patterns have been observed for ORF3 protein as well as its interaction with microtubules [77]. ORF3 protein upregulates the expression of glycolytic pathway enzymes via the stabilization of hypoxia-inducible factor 1 (HIF-1) and attenuates the cell mitochondrial death pathway [78], [79]. Sequence analyses indicate that ORF3 protein contains two hydrophobic domains in its N-terminus: D1 and D2, and two proline-rich domains toward its C-terminus: P1 and P2. The P2 domain has two PXXP motifs, which bind to many Src homology 3 (SH3) domains-containing cellular proteins and molecules [80], suggesting that the ORF3 protein is a regulatory protein involved in the modulation of cell signaling. For example, ORF3 protein binds CIN85, which competes with the formation of the growth factor receptor Cbl-CIN85 complex, leading to a delayed degradation of endomembrane growth factor and prolonged cell survival [81]. Furthermore, the PXXP motifs in ORF3 protein interact with tumor susceptibility gene 101 protein (TSG101) [82], [83], [84], an essential cellular factor of endosomal sorting complexes required for the transport (ESCRT) pathway. Additionally, ORF3 protein downregulates the toll-like receptor 3 (TLR-3) mediated nuclear factor kappa B (NF-κB) signaling via tumor necrosis factor receptor 1-associated death domain protein (TRADD) and receptor-interacting protein kinase 1 (RIP1) [85]. ORF3 protein also shares structural similarity to class I viroporins, and functions as a membrane ion channel to facilitate HEV particle release [86]. It has been reported that the palmitoylation of ORF3 protein is required to determine its subcellular localization and membrane topology [87]. Taken together, ORF3 protein is a multifunctional protein and plays a vital role in the HEV life cycle.

### 5.4 A novel ORF4 protein in genotype 1 HEV

A novel viral protein designated ORF4 protein, which is mapped within the ORF1 but translated from a different frame, is identified in most genotype 1 HEVs but not in other HEV genotypes [35]. The ORF4 protein has a molecular weight of approximately 20 kDa and is induced by ER stress [35], [88]. ORF4 protein interacts with multiple viral and host proteins, including Hel, RdRp, X, and eukaryotic elongation factor 1 isoform-1 (eEF1α1), and assembles a protein complex stimulating viral RdRp activity [35]. It appears that ectopic expression of ORF4 increases the genotype 3 HEV fitness in cell culture [89]. Nonetheless, many questions remain about the exact role of ORF4 in the HEV life cycle. For example, why does it exist only in genotype 1 HEV strains? Are there any functionally equivalent but not-yet-identified protein in other HEV genotypes?

## 6 Life cycle of HEV replication

**Fig. 5:**
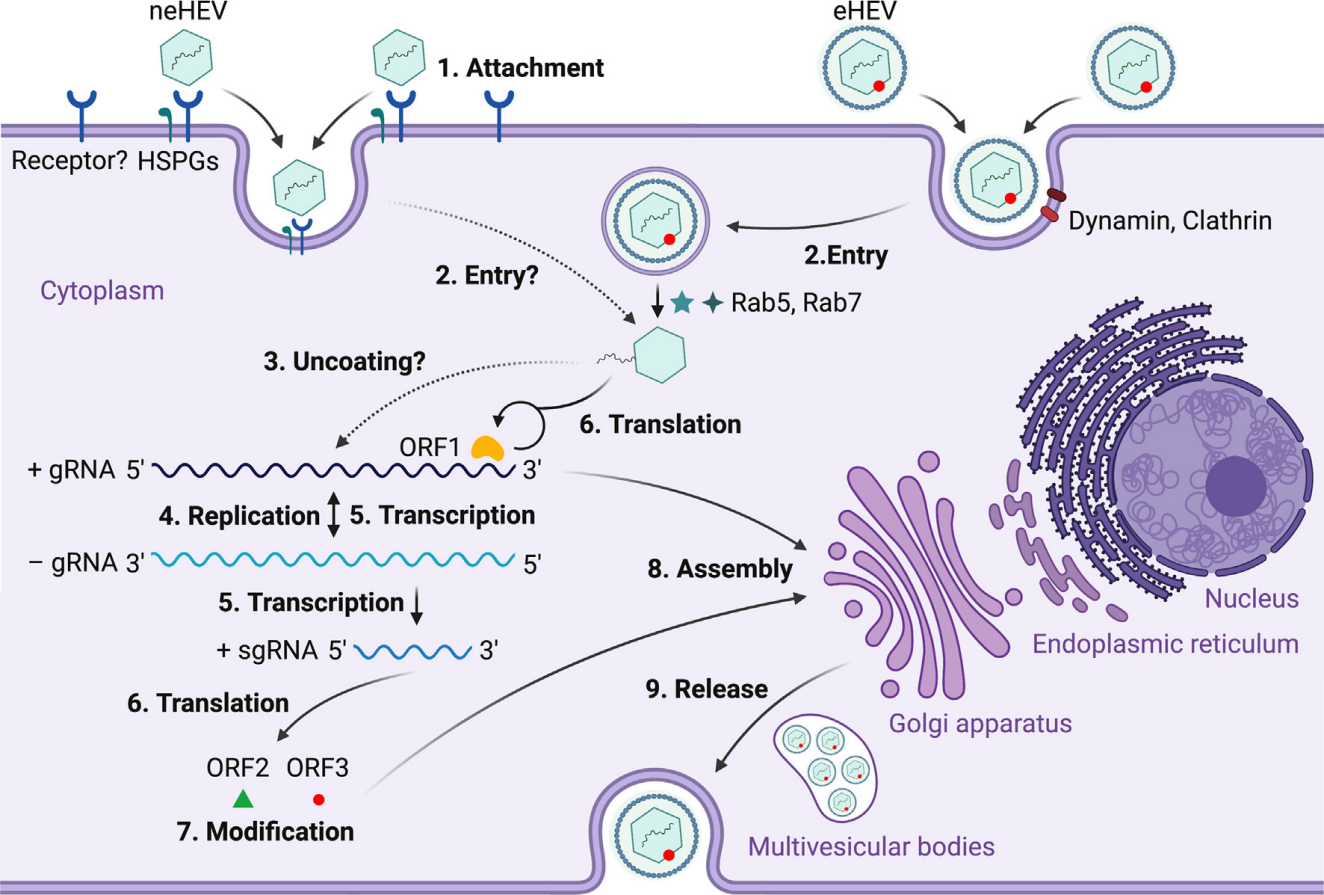
Proposed life cycle of hepatitis E virus (HEV). (1) Non-enveloped HEV (neHEV) particles bind to heparan sulfate proteoglycans (HSPGs) on the surface of liver cells and enter via an as yet unidentified specific cellular receptor; (2) Quasi-enveloped HEV (eHEV) particles enters liver cells via dynamin-dependent, clathrin-mediated endocytosis, which requires small GTPases Ras-related proteins Rab5 and Rab7; (3) The viral genomic RNA is released to cytosol after uncoating of capsid protein with an unknown process; (4) The viral genomic RNA directly serves as mRNA for ORF1 polyprotein translation, and also synthesizes a complementary negative-sense RNA to serve as a template for HEV replication; (5) The intermediate negative-sense RNA then serves as a template for transcription of full-length genomic as well as subgenomic mRNAs (sgRNAs); (6) More ORF1 polyproteins are translated from the full-length genomic RNA, and the ORF2 capsid protein and ORF3 multifunctional protein are translated from the sgRNAs; (7) ORF2 and ORF3 undergoes post-translational modifications such as glycosylation, phosphorylation, and palmitoylation; (8) ORF2 capsid protein self-assembles into virus-like particles (VLPs) and binds to newly synthesized positive-sense genomic RNA to form progeny HEV virions; (9) ORF3 regulates the host environment through interaction with a number of cellular proteins to promote viral replication and virion secretion. Specifically, ORF3 binds to TSG101 involved in the ESCRT pathway, facilitating the budding of nascent virions into multivesicular bodies, which fuse with the plasma membrane, and the virions are released from the liver cells either into the bloodstream as eHEV or in the bile duct as neHEV. The figure is created with Biorender.com.

The HEV life cycle remains poorly understood to date, largely due to the lack of an efficient cell culture system to propagate the virus. As a fecal-orally transmitted virus, HEV first enters the host via the gastrointestinal tract and replicates in intestinal epithelial cells. Subsequently, the virus enters the bloodstream via viremia and reaches its target organ, the liver [90]. Heparan sulfate proteoglycans (HSPGs) are important for HEV cellular binding, but a specific cellular receptor for HEV attachment is still not identified [91] (Fig. 5). It is believed that the eHEV and neHEV exploited distinct virus entry mechanisms: eHEV enters cells via a dynamin-dependent, clathrin-mediated endocytosis, which requires small GTPases Ras-related proteins Rab5 and Rab7, and the lipid membrane is degraded by a lysosomal protein Niemann-Pick disease type C1 (NPC1). The knowledge regarding neHEV entry mechanism is scarce [92]. After an unknown uncoating process of HEV capsid, the viral genomic RNA directly serves as mRNA for ORF1 polyprotein translation, which produces a number of functional enzymes or domains as outlined above. The viral replicase RdRp synthesizes a complementary negative-sense RNA to serve as a template for HEV replication and transcription of the sgRNA, which is responsible for translating ORF2 protein and ORF3 protein. The negative-sense RNA has been detected in livers and extrahepatic tissues of various animals experimentally infected with HEV [90]. It has been reported that ORF1 polyprotein localizes to ER membranes, which probably is the site of HEV replication [45], [51]. The secreted form ORF2^S^ undergoes post-translational modifications and acts as immune decoys, whereas the capsid-associated form ORF2^C^ self-assembles to VLPs, and packages genomic RNA to progeny HEV virions [34]. ORF3 protein interacts with microtubules and multiple host cellular proteins in modulating the host environment for HEV replication [73], [77], [87]. Importantly, ORF3 protein binds to TSG101 involved in the ESCRT pathway, facilitating the budding of nascent virions into multivesicular bodies [83]. Finally, the multivesicular bodies fuse with the plasma membrane, and the virions are released from liver cells either into the bloodstream wrapped with a lipid membrane (eHEV), or in the bile duct where the quasi-envelope is degraded by bile salts [22]. In-depth reviews on interactions between viral and host cell factors during HEV infection can be found elsewhere [93]. The underlying molecular mechanisms in several steps of HEV life cycle, especially the entry, uncoating, assembly and release steps, remain poorly understood. Recent advances in developing cell lines that support more efficient HEV replication will aid mechanistic studies of HEV life cycle, which will be crucial for developing HEV-specific antiviral drugs in the future [94], [95], [96].

## 7 Summary and outlook

Significant advances have been made in many aspects of HEV structural and molecular biology, including determination of the 3D structure of HEV VLPs, identification of quasi-enveloped form of HEV virions in blood, discovery of two distinct forms of capsid protein with different functions, identification of four regulatory *cis-reactive* elements, and establishment of more efficient cell culture systems for HEV propagation. These novel discoveries help understand the underlying mechanisms of HEV replication and pathogenesis. However, significant scientific gaps remain for this important but extremely-understudied pathogen. Many novel strains of HEV still cannot be efficiently propagated, and development of more efficient HEV cell culture system should still be a priority so that we can delineate the virus replication cycle and develop cost-effective vaccine. Many critical steps in the HEV life cycle remain poorly understood, which hinders the development of HEV-specific antivirals. The structural and functional relationship of HEV genes and their roles in HEV pathogenesis especially cross-species infection also warrant further in-depth study. The HEV vaccine Hecolin is approved for use only in China, it will be important to determine its protective efficacy against the diverse genotypes of HEV, especially the emerging zoonotic HEV strains.

## CRediT authorship contribution statement

**Bo Wang:** Conceptualization, Investigation, Writing - original draft. **Xiang-Jin Meng:** Conceptualization, Supervision, Writing - review & editing.

## Declaration of Competing Interest

The authors declare that they have no known competing financial interests or personal relationships that could have appeared to influence the work reported in this paper.
